# The Amick Quack Company

**Published:** 1894-06

**Authors:** 


### THE AMICK QUACK COMPANY.

Doctors over the country have been well sampled with literature from the great Amick Chemical Cure Company, containing some very specious invitations to try their medicines. The company itself is made up of one safe-manufacturer, one newspaper agent, and one doctor (formerly an eye and ear specialist). They have been suing several parties lately, journals and individuals, but have always been defeated. Only a few weeks ago they sued for $25,000 damages, but subsequently compromised for twenty-five dollars. Down in Texas the Amick Chemical Combined Quack Cure Company seem to be in rather bad odor. We congratulate the Texas Medical Association on the following resolutions passed at the last meeting:

WHEREAS, A secret nostrum manufacturing concern known as the Amick Chemical Company, located at Cincinnati, Ohio, have for the past several months flooded the United States with circulars of printed matter directed to almost every physician throughout the country, and to many thousands of private persons, claiming to cure tubercular consumption and some other diseases by the use of a secret compound known as the Amick Chemical Cure; and

WHEREAS, This concern have by fraud made illegitimate use of the Associated Press dispatches, and have used the names of men eminent in the profession, without their consent, for the purpose of advertising their said secret nostrum, and for the purpose of deceiving the people throughout the country; and

WHEREAS, The claim has been made and circulated broadcast, that this secret nostrum has the indorsement of the members of this Association, and of the regular profession generally, as well as the reputable medical journals throughout the country; and

WHEREAS, These sharpers, engaged in the manufacture of this secret nostrum, with consummate skill and shrewd money-making judgment, have undertaken, with unblushing effrontery, to use the medical profession in the distribution and sale of their nostrums by refusing to send their so-called chemical cure to patients except through the medical attendant; therefore be it

*Resolved*, By the Texas State Medical Association, that we denounce the statement that the so-called chemical cure has the indorsement of this Association, or any reputable member of it, as false; and the peculiar and reprehensive methods of advertising adopted by this concern, and the attempt to use the members of the profession in the distribution and sale of a secret nostrum, are hereby condemned and denounced, and that we advise the members of this Association, and the profession generally throughout the country, to refuse their aid to this concern in the distribu-

**tion and sale of their secret nostrum, which should be treated, in all respects, as other secret and patent medicines.**

A few weeks ago we had the pleasure of declining to advertise the Amick Company, and we think every other journal should do likewise.

